# The impact of neoadjuvant chemotherapy on immunohistochemistry and molecular subtype in breast cancer: A retrospective analysis from Oman

**DOI:** 10.5339/qmj.2026.3

**Published:** 2026-03-03

**Authors:** Mustafa Talib Al-Ani, Asila Rashid Al Ismaili, Nahad Al-Mahrouqi, Hasan Al-Sayegh, Khalid Al Baimani, Hind Saif Ali Alshahi, Arti Trehan, Zaid Al-Ishaq, Marwa Youssef, Asma Naaz Nadaf, Adil Aljarrah Alajmi

**Affiliations:** 1 Department of Surgery, Sultan Qaboos Comprehensive Cancer Care and Research Centre, University Medical City, Oman; 2 Research Laboratories Department, Sultan Qaboos Comprehensive Cancer Care and Research Centre, University Medical City, Oman; 3 Department of Medicine, Sultan Qaboos Comprehensive Cancer Care and Research Centre, University Medical City, Oman; 4 Department of Pathology, Sultan Qaboos Comprehensive Cancer Care and Research Centre, University Medical City, Oman *Email: mustafa.talib.2@gmail.com

**Keywords:** Neoadjuvant therapy, breast neoplasms, Estrogen receptors, Progesterone receptors, ErbB-2 receptors

## Abstract

**Background:**

Neoadjuvant chemotherapy (NACT) is used in breast cancer (BC) to downsize and downstage the tumor before surgery. Different studies were conducted looking into the alterations in the immunohistochemistry after NACT, which may alter the adjuvant treatment. Our study aimed to assess the changes in immunohistochemistry biomarker status (estrogen receptor [ER], progesterone receptor [PR], and human epidermal growth factor receptor 2 [HER2]) in BC cells after administration of NACT at a single institution in Oman.

**Methods:**

We conducted a retrospective cross-sectional study on patients with BC with residual disease post-NACT in a single institution and studied immunohistochemistry status changes before and after NACT. We used the McNemar test to evaluate the receptor changes, and logistic regression to assess the effect of risk factors on receptor status change.

**Results:**

All biomarkers changed after NACT, with a tendency for BC cells to lose PR and HER2 expression and gain ER expression. The immunohistochemistry changes were 36/114 (31.6%) in PR, 7/114 (6.1%) in ER, and 11/114 (9.6%) in HER2. BC subtypes changed in 4/114 (3.5%) of HER2+ve, 10/114 (8.8%) of hormone positive (ER or PR) with HER2 overexpression (HER2+ve/HR+ve), 3/114 (2.6%) in hormone positive with HER2 negative (HR+ve), and 3/114 (2.6%) triple-negative BCs. These changes resulted in adjuvant treatment adjustments in 9/110 (8.18%) patients with residual disease.

**Conclusion:**

Changes in the expression of all immunohistochemistry biomarkers and BC subtypes occurred after NACT, which led to changes in adjuvant treatment in specific cases.

## 1. INTRODUCTION

Breast cancer (BC) is the most prevalent cancer in Oman, with 277 cases diagnosed in 2020, representing 14.3% of all cancer diagnoses, with an overall increasing trend in the last two decades.^1,2^ Patients with BC present with advanced stages of disease and at a relatively young age.^3^ Furthermore, patients with BC had improved relapse-free survival and overall survival, which can be explained by the use of different treatment modalities, including evidence-based neoadjuvant chemotherapy (NACT) protocols.^4^

Since 2006, NACT, often including targeted therapy, has become standard for primary BC. Its use aims to downsize tumors, enabling more conservative surgery, reducing mortality, and providing prognostic information as assessed by pathological complete response (pCR). Changes in BC immunohistochemistry biomarkers were reported with different frequencies across the literature, affecting progesterone receptors (PR), estrogen receptors (ER), and human epidermal growth factor receptor 2 (HER2).^5–10^

For those reasons, NACT is regularly used for patients with BC in our institution. The decision regarding the type of neoadjuvant therapy, as well as adjuvant treatment, is based on the stage, as well as the assessment of the immunohistochemistry biomarkers status tested on the biopsy tissue and surgical specimen tissue, respectively.^11^ Multi-gene assays are also utilized to decide the treatment of patients with different luminal diseases.^12^ The objectives of the study were to find the rate of change of each tumor biomarker between pre-NACT on biopsy testing and post-NACT testing of the surgical specimen, the rate of change of the molecular subtype after NACT, and the change of adjuvant treatment provided to the patients in which these immunohistochemistry biomarker changes were found. The routine retesting of immunohistochemistry markers after NACT is not consistently performed in clinical practice, and there is a significant gap in the literature regarding the clinical value and impact of such retesting. We hypothesized that immunohistochemistry biomarker and subtype changes occur frequently enough post-NACT to justify routine retesting. Therefore, our study aimed to address this gap by investigating the results of immunohistochemistry retesting after NACT, to provide evidence to inform clinical decision-making.

## 2. METHODOLOGY

### 2.1 Patients

We conducted a retrospective cohort study on patients with BC who received NACT and then underwent surgical excision between January 2021 and October 2024. Electronic data were collected using the SAP Hospital Information System (HIS) of Sultan Qaboos Comprehensive Cancer Care and Research Centre (SQCCCRC) after receiving authorization from the ethics committee at SQCCCRC (CCCRC-28-2023) on September 17, 2023.

#### 2.1.1 Inclusion criteria

All patients with BC who had NACT followed by surgery and had immunohistochemistry retesting after surgery on the residual tumor.

#### 2.1.2 Exclusion criteria

Patients who did not undergo NACT, did not undergo operation, developed pCR after surgery, and those who lost follow-up during the treatment phase.

**Figure 1.**
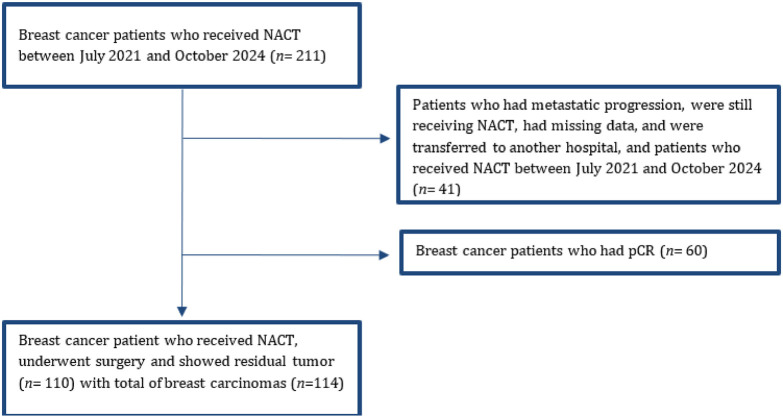
Flow chart of sample selection of the study.

A total of 211 patients received NACT after being diagnosed with BC between July 2021 and October 2024. Patients who did not undergo surgery (metastatic progression, refused surgery, were still receiving NACT, transferred to another hospital, and patients with missing data; *n* = 41) and those who had pCR (*n* = 60) were excluded. In total, 110 patients met the inclusion criteria, with four patients having bilateral disease, leading to 114 breast carcinomas included (Figure 1). No sampling method was applied; instead, we included all eligible patients consecutively. Patients’ demographics, clinical notes, clinical TNM staging, histology type, histology grade, types of NACT, and the biomarker status before and after NACT were collected for each patient.

The patient evaluation was done clinically by physical examination, radiologically by mammography, ultrasonography, and breast MRI with Gadolinium contrast for local disease evaluation and positron emission tomography or computed tomography (CT) for metastasis evaluation. Histological samples were taken by core needle biopsy from the breast, and fine needle aspiration cytology from the axilla, where indicated, before NACT, and surgical specimens were sent for histopathological examination after NACT. Immunohistochemistry biomarkers: ER, PR, and HER2 were done before and after surgery. However, the Ki-67 was not retested after NACT in the surgical specimens, as it is not currently endorsed to be routinely retested to guide post-NACT management.^12^ BC subtypes: The patients were classified into four subtypes according to their immunohistochemistry status as: (1) hormone negative with HER2 overexpression (HER2+ve), (2) hormone positive (ER or PR) with HER2 overexpression (HR+ve/HER2+ve), hormone positive (ER or PR) with no HER2 overexpression (HR+ve), hormone negative with no HER2 overexpression or triple-negative (TN), and these subtypes were compared before and after NACT as displayed in Table 3. pCR was considered as the absence of invasive carcinoma in the breast tissue and the absence of compromised lymph nodes (ypT0N0). The presence of an in-situ component in the breast tissue (pTis N0) was considered as pCR in accordance with the TNM staging system (8th edition).^13,14^

### 2.2 Pathological analysis

Tumor grade was graded according to the Nottingham Histologic Score system. The immunohistochemical analysis of the ER and PR was performed using the EP-1 antibody and PgR 1294 antibody, respectively, in Dako Omnis, adhering to the protocols, and the interpretation of both receptors was performed with the Allred scoring protocol. HER2 pathway antibody was used for HER2 analysis, which was performed in Ventana Roche, and the interpretation was carried out using ASCO CAP guidelines.^15^ Additionally, Ki67 proliferation index analysis was also carried out based on the positive tumor nuclei per 100 tumor cells. The results were expressed as a percentage.

### 2.3 Statistical analysis

Descriptive statistics were performed to summarize the clinical characteristics of the study population. Means, standard deviations, medians, and interquartile ranges were reported for the continuous variables, and frequencies and percentages were reported for the categorical variables. We used the Fisher exact test and the Wilcoxon rank sum test to examine differences between the categorical and continuous variables, respectively. We performed the McNemar test to evaluate changes in paired categorical outcomes (changes in receptor status between pre- and post-NACT). Logistic regression was used to examine the effect of risk factors on binary outcomes (any change post-treatment in the ER, PR, HER2 status from positive to negative or vice versa). Univariable logistic regression models were constructed to evaluate the effect of each predictive factor. All statistical analyses were performed using the R statistical software; two-sided *P*-values ≤0.05 were considered statistically significant.

## 3. RESULTS

### 3.1 Study demographics

The total number of patients that fulfilled the inclusion criteria was 110, with four of them having bilateral BCs, giving a total of 114 carcinomas that were included in the study. One hundred and nine of the patients were females, and only one patient was a male. Ages ranged between 25 and 80 years old (median, 45 years), and with an average body mass index (BMI) of 23.3. Table 1 shows the clinical, histopathological, and biological characteristics of pre- and post-NACT carcinomas that were included in this study. The pre-NACT histology of most of the carcinomas was non-specific type invasive ductal carcinoma, comprising 104 (91.2%) cases, whereas the remaining carcinomas, 10 (8.8%), had histology of invasive lobular carcinoma. Moreover, 70 (61.4%) cases had grade II, 39 (34.2%) cases had grade III, while only 5 (4.4%) cases had grade I.

Regarding the type of NACT, 75 (65.8%) cases received doxorubicin/cyclophosphamide followed by taxane. Furthermore, 16 (14%) cases received taxane/carboplatin/trastuzumab/pertuzumab, 12 (10.5%) cases received keynote 522, and five (4.4%) cases received doxorubicin, cyclophosphamide, followed by taxane/trastuzumab/pertuzumab. Moreover, two (1.1%) cases received doxorubicin/cyclophosphamide followed by taxane/carboplatin/trastuzumab/pertuzumab, two (1.8%) cases received hormonal therapy, and two (1.8%) cases received taxane/trastuzumab/pertuzumab. Table 2 describes the type of NACT based on the immunohistochemistry subtype and divides the patients into two groups based on metastasis on the clinical TNM staging: Neoadjuvant without metastasis at diagnosis and pseudo-neoadjuvant for the patients with metastatic disease at diagnosis.

### 3.2 Changes in receptor status

Changes in ER, PR, and HER2 receptor status (from negative to positive or from positive to negative) were noted in 7/114 (6.1%), 36/114 (31.6%), and 11/114 (9.6%) cases, respectively. Table 3 showed the changes in receptor status pre- and post-NACT, where it was found that PR had the highest frequency of change, followed by HER2 receptors, then ER. PR status changed from positive to negative in 31 (27.2%) samples, while five (54.4%) samples shifted from negative to positive. Furthermore, HER2 receptor status changed from positive to negative in nine (7.9%) samples, while two (1.8%) samples changed from negative to positive. ER receptor status changed in two (1.8%) samples from positive to negative, while five (4.4%) samples changed from negative to positive. The total number of tumors that displayed any change (negative to positive or vice versa) in ER, PR, and/or HER2 was 46 (40.4%).

### 3.3 Changes in molecular subtype status

In the majority of cases, 75 (65.8%), at diagnosis, presented with HR+ve, while 19 (16.7%) were HR+ve/HER2+ve, 15 (13.2%) cases presented as TN, and five (4.4%) cases presented with HER2+ve status. The highest stability of subtypes from change post-NACT was found in HR+ve and TN tumors, with 72 (63.1%) and 12 (10.5%) cases, respectively. While HER2+ve changed the subtype in 4/114 (3.5%) cases, and HR +ve/HER2 +ve changed in 10/114 (8.8%). Total molecular subtype changes happened in 20/114 (17.5%) carcinomas (Table 4).

### 3.4 Changes in adjuvant treatment

Overall, 9/110 (8.18%) patients had adjustments in their adjuvant treatment by adding one of the following: (1) hormonal therapy for patients who displayed negative to positive expression of HR receptors in six patients, (2) anti-HER2 therapy for patients who displayed negative to positive expression of HER2, seen in two patients, and (3) capecitabine was added for one of the two patients who switched subtype into TN BC.

### 3.5 Predictive univariable models of the changes in receptor status

Predictive univariable models to predict any change in receptor status (any change in ER, PR, HER2 status from positive to negative or vice versa) revealed a statistically significant result (*P*-value: 0.0044) for HER2+ve cases as opposed to HER2-ve cases (Table 5). While this finding is statistically significant, it must be interpreted with caution because the logistic regression output for this variable was associated with wide confidence intervals, suggesting limited precision and potential underpowering of the study. Other variables, including age, sex, BMI, T stage, N stage, metastasis (M), histopathology, grade, ER status, PR status, KI67, and type of chemotherapy, did not show statistical significance.

## 4. DISCUSSION

Immunohistochemistry biomarker changes in BC cells can be detected due to both spatial and temporal heterogeneity. While spatial heterogeneity acts as a non-avoidable limitation to our study, because it describes the different cellular characteristics at different locations inside the same tumor, the temporal heterogeneity however, being caused by the cancer cells adjusting their phenotype due to multiple factors such as environmental factors like NACT, is a good variable to study as it may lead to adjustments in adjuvant treatment helping to tailor it for different patients individually.^16–19^ While there is no strong evidence base confirming the importance and significance of these changes in the adjustments in adjuvant treatment and overall survival according to current guidelines, multiple studies have examined this issue. For that reason, we investigated our data retrospectively to look for the frequency and type of these changes as well as to see how often they caused adjustments in the adjuvant therapies.^11,20,21^ In this study, we conducted a reassessment of ER, PR, and HER2 after NACT, and showed that forty percent of tumors had a change in the status of at least one of these biomarkers, shifting from positive to negative or vice versa. PR status demonstrated the highest frequency of change in our study, in almost one third of cases, which was statistically significant (*P*-value < 0.0001), and tended to lose expression. In the literature, PR status change varied between (14.5%–30%), with a tendency for a loss of expression as reported by Candás et al. Al-Saleh et al. and Gahlaut et al.^20,22,23^ On the contrary, Coiro et al. found that PR change had a tendency for positive expression post-NACT. Our study findings were concordant with the published literature regarding PR having the highest percentage of change.^24^ HER2 receptors were the second most affected by status change in our study, with a loss of expression tendency. In other studies, the change in HER2 receptor status ranged between 7% and 16.5%, with a tendency to lose expression, as found by our study.^20,22–24^

Our findings, when compared to the large meta-analysis by Zhang et al. revealed both similarities and differences in immunohistochemistry conversion rates. Specifically, the percentage of change in ER status in our study was 6.1%, which was substantially lower than the 18.1% reported by them. Conversely, the PR and HER2 conversion rates were higher in our study (31.6% and 9.6%) versus (26.6% and 5.4%) by the meta-analysis, respectively. Similar to their findings, the immunohistochemistry changes of BC cells after NACT displayed more ER expression and less PR expression, while in our data, HER2 displayed loss of expression, but Zhang et al. showed equal rates of loss and gain of expression.^25^

In the literature, different findings were reported, with some reporting negative to positive, while others reported positive to negative. The percentages of change that were reported ranged between 5.6% and 23%.^20^ Regarding immunohistochemistry subtypes, the results of this study revealed notable shifts in BC immunohistochemistry subtypes following NACT. The dominance of HR+ve cases pre-NACT remained mostly stable after NACT. Moreover, a significant change observed is the transition from HER2+ve/HR+ve subtypes to HR+ve, which suggests that NACT may contribute to a reclassification of tumors, possibly due to the response of the tumor or changes in expression of the receptor during treatment. Only 20% of TN BC cases transitioned to HR+ve after NACT, while the remaining cases remained unchanged. This implies that while a few TN tumors may have experienced receptor conversion, the majority maintained their TN status, emphasizing the ongoing difficulty in treating these aggressive and treatment-resistant cancers. In comparison, Lim et al. reported that among 322 patients with BC after NACT, 89.2% of HR+ve patients remained HR+ve after NACT, which was concordant with our results.^26^ On the other hand, Lim et al. found that 10.3% of HR+ve tumors showed a conversion into TN tumors, whereas we found a smaller percentage in our study. Our study also showed a smaller percentage of TN BC cases changing to other subtypes.^26^ Changes in treatment were made in 9/110 (8.18%) of the total patients that had residual disease, compared to 3.8% found by Coiro et al.^24^ and 7.3% was reported by Candás et al.^20^ This affected patients with new post-NACT expression of Hormone receptors or HER2 through addition of targeted therapy, and for tumors switching the immunohistochemistry subtype to TN post-NACT the addition of Capecitabine was made, for this reason we are suggesting that routine retesting post NACT is further studied with more future research evaluating its cost effectiveness. Predictive models to predict any change in receptor status (any change in ER, PR, HER2 status from positive to negative or vice versa) revealed a statistically significant result (*P*-value = 0.0044) for HER2+ve cases as opposed to HER2-ve cases. While all published studies investigating the immunohistochemistry changes post-NACT have reported alterations, the frequency and direction of these changes (e.g., from positive expression to loss of expression or vice versa) have varied across the literature. This variability could stem from multiple factors, such as tumor heterogeneity leading to biases in sample acquisition, technical factors in immunohistochemistry testing, biological variability in tumor cell response to NACT, different sample sizes, and potential unmeasured confounding variables. Our study was particularly important because it contributed to the existing literature by reporting findings from a Middle Eastern population. Methodologically, it minimized selection bias through the inclusion of all consecutive cases and employed standardized immunohistochemistry testing and retesting protocols across the samples. Understanding the factors driving receptor status changes is essential. To this end, we performed predictive modeling to evaluate the impact of various risk factors on immunohistochemistry alterations following NACT. We hope that our findings will enhance understanding of immunohistochemistry changes post-NACT and support more informed, evidence-based clinical decision-making.

### 4.1 Limitations

Our study has several limitations, starting with its retrospective nature, which restricts the ability to infer definitive causality of the immunohistochemistry changes. Additionally, the change in immunohistochemistry status found post-NACT could be partially attributed to unavoidable sampling error because of the tumor’s spatial heterogeneity. Furthermore, the analysis was limited by sample size, which led to wide confidence intervals for some factors of the logistic regression models, reflecting limited statistical power and reducing the precision of estimates. Finally, the lack of Ki67 data limited our analysis, as this proliferation index is crucial for tumor subtype classification, particularly in differentiating between luminal A and B breast cancer.

## 5. CONCLUSION

In summary, our study showed that ER, PR, and Her2 expression changes after NACT were 6.1%, 31.6%, and 9.6%, respectively, while the molecular subtypes changed in 17.5% that occurred across all BC intrinsic subtypes. Due to these changes, the Adjuvant treatment was adjusted in 8.18% of patients with BC. Therefore, biomarker retesting after NACT is important to assist in choosing and tailoring the adjuvant therapy plan.

## CONFLICT OF INTEREST

The authors declare that there is no conflict of interest.

## Figures and Tables

**Table 1.** The clinical, histopathological, and biological characteristics of pre- and post-NACT patients with residual disease (*n* = 114 breast carcinomas).

Variable	
Age, median [range] ± SD	45.0 [25.0–80.0] ± 10.3	
BMI, mean [range] ± SD	23.0 [13.59–38.41] ± 4.8	
**Gender**	***n***	**Percentage**
Female	113	99.1%
Male	1	0.9%
**T**		
1	11	9.6%
2	67	58.8%
3	19	16.7%
4	17	14.9%
**N**		
0	7	6.1%
1	63	55.3%
2	8	7.0%
3	36	31.6%
**M**		
0	102	89.5%
1	12	10.5%
**Histology pre-NACT**		
IDC	104	91.2%
ILC	10	8.8%
**Grade pre-NACT**		
1	5	4.4%
2	70	61.4%
3	39	34.2%
**Estrogen receptors**		
ER+	92	80.7%
ER−	22	19.3%
**Progesterone receptors**		
PR+	88	77.2%
PR−	26	22.8%
**Human epidermal growth factor receptor 2**		
HER2+	24	21%
HER2−	90	78.9%
**Types of neoadjuvant chemotherapy**		
Doxorubicin/cyclophosphamide followed by taxane	75	65.8%
Taxane/carboplatin/trastuzumab/pertuzumab	16	14.0%
KEYNOTE 522	12	10.5%
Doxorubicin/cyclophosphamide followed by taxane, trastuzumab plus pertuzumab	5	4.4%
Doxorubicin/cyclophosphamide followed by taxane/carboplatin/trastuzumab/pertuzumab	2	1.8%
Hormonal	2	1.8%
Taxane/trastuzumab/pertuzumab	2	1.8%

**Table 2.** Chemotherapy regimens according to immunophenotype.

Immunophenotype	Neoadjuvant therapy (*n* = 102)	*n* (%)
**HR+ve, *n* = 68**	Doxorubicin/cyclophosphamide followed by taxane	67 (98.5%)
	Hormonal	1 (1.5%)
**HER2+ve, *n* = 4**	Taxane/Carboplatin/trastuzumab/Pertuzumab	4 (100.0%)
**HR+ve/**	Taxane/Carboplatin/trastuzumab/Pertuzumab	11 (64.7%)
**HER2+ve, *n* = 17**	Doxorubicin, cyclophosphamide, followed by taxane, trastuzumab/pertuzumab	4 (23.5%)
	Doxorubicin/cyclophosphamide followed by taxane/Carboplatin/trastuzumab/Pertuzumab	2 (11.8%)
**TN, *n* =13**	Doxorubicin/cyclophosphamide followed by taxane	2 (15.4%)
	KEYNOTE 522	11 (84.6%)
**Immunophenotype**	**Pseudo-neoadjuvant therapy (*n* = 12)**	***n* (%)**
**HR+ve, *n* = 7**	Doxorubicin/cyclophosphamide followed by taxane	6 (85.7%)
	Hormonal	1 (14.3%)
**HER2+ve, *n* = 1**	Taxane/trastuzumab/pertuzumab	1 (100.0%)
**HR+ve/HER2 +ve, *n* = 2**	Taxane/Carboplatin/trastuzumab/Pertuzumab	1 (50.0%)
	Doxorubicin, cyclophosphamide, followed by taxane, trastuzumab/pertuzumab.	1 (50.0%)
**TN, *n* = 2**	Doxorubicin/cyclophosphamide followed by taxane	1 (50.0%)
	KEYNOTE 522	1 (50.0%)

**Table 3.** Change in the receptor status pre- and post-NACT treatment (total *n* = 114).

Receptor	Status		*n*	percentage	*P*-value
	Pre-NACT	Post-NACT			
**ER, *n* (%)**					
Unchanged	+	+	90	78.9%	0.45
107 (93.8%)	−	−	17	14.9%	
Changed	+	−	2	1.8%	
7 (6.1%)	−	+	5	4.4%	
**PR, *n* (%)**					
Unchanged	+	+	57	50%	<0.0001
78 (68.4%)	−	−	21	18.4%	
Changed	+	−	31	27.2%	
36 (31.6%)	−	+	5	4.4%	
**HER2, *n* (%)**					
Unchanged	+	+	15	13.2%	0.07
103 (90.3%)	−	−	88	77.1%	
Changed	+	−	9	7.9%	
11 (9.6%)	−	+	2	1.8%	
Any change (ER, PR, and/or HER2)	+/−	+/−	46	40.4%	Not applicable

**Table 4.** Change in subtype, pre- and post-NACT (*n* = 114).

Pre-NACT	*n* (%)	Post-NACT			
		HER2 +	HR+/HER2 +	HR +	TN
**HER2 +**	5 (4.4%)	1 (0.9%)	3 (2.6%)	1 (0.9%)	-
**HR+/HER2 +**	19 (16.7%)	2 (1.8%)	9 (7.9%)	7 (6.1%)	1 (0.9%)
**HR+**	75 (65.8%)	-	2 (1.8%)	72 (63.1%)	1 (0.9%)
**TN**	15 (13.2%)	-	-	3 (2.6%)	12 (10.5%)

**Table 5.** Predictive univariable models of the changes in receptor status (*n* = 114).

Model	OR (95%CI)	*P*-value
Age (one year increase)	1.02 (0.98–1.06)	0.2471
Male versus female	0.48 (0.003–9.27)	0.642
BMI (one unit increase)	0.95 (0.85–1.04)	0.2839
**T stage**		
2 versus 1	1.34 (0.37–5.5)	0.6675
3 versus 1	0.63 (0.12–3.22)	0.5642
4 versus 1	1.56 (0.33–7.88)	0.5774
**N stage**		
1 versus 0	3.95 (0.62–76.93)	0.2163
2 versus 0	6 (0.6–143.41)	0.1652
3 versus 0	4.8 (0.72–95.6)	0.1655
**M stage**		
M1 versus M0	1.06 (0.3–3.56)	0.9218
**Histopathology**		
ILC versus IDC	0.98 (0.24–3.66)	0.9811
**Grade**		
2 versus 1	2.83(0.39–56.88)	0.3633
3 versus 1	2.78(0.37–57.17)	0.3795
**Immunohistochemistry**		
ER + versus −	0.97 (0.38–2.57)	0.9526
PR + versus −	1.37 (0.56–3.53)	0.4984
HER2 + versus −	4 (1.58–10.87)	0.0044
KI67 (≥20 versus <20)	0.23 (0.03–1.26)	0.1131
**Type of chemotherapy**		
Pseudo-neoadjuvant versus adjuvant	1.06 (0.3–3.56)	0.9218
